# Nursing Homes Impact Hospice Care for Connecticut Medicaid Decedents with Serious Illnesses

**DOI:** 10.1093/geroni/igaf122.1697

**Published:** 2025-12-31

**Authors:** Ellis Dillon, Chae Man Lee, Doreek Charles, Wenqi Gan, Germine Soliman, Julie Robison

**Affiliations:** University of Connecticut, Farmington, Connecticut, United States; University of Connecticut, Farmington, Connecticut, United States; University of Connecticut, Farmington, Connecticut, United States; University of Connecticut, Farmington, Connecticut, United States; University of Connecticut, Farmington, Connecticut, United States; University of Connecticut, Farmington, Connecticut, United States

## Abstract

Hospice care is underutilized in nursing home (NH) populations for unclear reasons. This mixed methods study analyzed Connecticut Medicaid, Medicare, Minimum Data Set, and NH characteristics data; and thematically analyzed 14 in-depth interviews with hospice and NH professionals about hospice care in NHs. The cohort included 39,633 Medicaid-insured decedents with serious illnesses in Connecticut from 2017-2023. Overall, 24,512 (61.9%) individuals had NH stays and 18,428 (46.5%) received hospice care ≤ 6 months of death, 7,265 (39.4%) with short hospice length of stay (≤ 7 days). Individuals with NH stays (versus without) had higher rates of hospice use (49.0% versus 42.4%). However, in multivariable analysis long-term NH stays were associated with reduced odds of hospice use. Among those with NH stays, the odds of receiving hospice care were higher for individuals with stays at NHs that were part of a chain, had an Alzheimer’s unit, and had lower CMS quality ratings. Interviews revealed barriers to hospice use including the focus on rehabilitation, policy disincentives, NH workflows/priorities, and staffing: “They miss the fact that someone’s declining if it’s a slow decline or if there’s a lot of different nurses caring for residents.” Facilitators include acute care use triggering hospice discussions, hospice benefitting NH patients and staff, and leveraging care planning meetings for hospice education/discussions. Overall, Connecticut Medicaid individuals with NH stays had lower odds of receiving hospice care, but certain NH characteristics, workflows, education, and policies influenced the likelihood of receiving hospice care and could be mechanisms for policy and practice change.

